# Satellite-driven assessment of methane trends, seasonal variability, and emission hotspots in Botswana’s Central and Ngamiland Regions

**DOI:** 10.1007/s10661-025-14609-y

**Published:** 2025-09-24

**Authors:** Boitshwarelo Lorato Masocha, Paidamwoyo Mhangara

**Affiliations:** School of Geography, Archaeology and Environmental Studies, University of the Witwatersrand, Johannesburg, Gauteng South Africa

**Keywords:** Methane emissions, Sentinel-5P, Botswana, Seasonal trends, Anomalies, Wetlands, Climate mitigation

## Abstract

This study investigates methane, a potent greenhouse gas linked to global warming, using high-resolution satellite data from the Sentinel-5P TROPOMI between 2020 and 2023. The research analyzed trends, seasonal changes, and emission hotspots in two different regions of Botswana: Central and Ngamiland. These areas have different methane sources, including human activities and natural processes. The results indicate significantly varying mean CH_4_ concentrations ranging from 1750 to 1900 ppb. The Central region has greater variability in concentrations with values between 1832.93 and 1888.86 ppb, compared to Ngamiland which has CH_4_ distribution ranging from 1837.92 to 1872.99 ppb. Increased summer CH_4_ levels are attributed by higher temperatures and wetter conditions that stimulate methanogenesis. In winter, levels lower due to cooler temperatures and dry conditions that reduce biological activity. Time-series analysis indicated an upward trend in both regions, suggesting an increase in emissions. The Mann–Kendall tests these significant upward trends in both areas. In late 2021, both regions showed positive anomalies, indicating potential methane risks in subsequent months/years. The ongoing rise in methane concentrations poses serious environmental and health threats to Botswana without effective action. Thus, this study supports focused mitigation strategies and seasonal management that aligns with Sustainable Development Goal 13 (Climate Action). This approach provides policymakers and environmentalists with valuable insights to combat climate change and support healthier, more sustainable communities.

**Supplementary Information:**

The online version contains supplementary material available at 10.1007/s10661-025-14609-y.

## Introduction

Methane (CH₄) is known as the second strongest (Gondwe & Masamba, 2014) contributor to anthropogenic greenhouse gas radiative forcing after carbon dioxide (Skeie et al., 2023). CH_4_ has a stronger global warming potential (Pachauri et al., 2014; Trenchev et al., 2023a, 2023b) per molecule and a relative short atmospheric lifetime of about 10 years (Stecher, 2024). The global mean methane concentration reached 1908 parts-per-billion (ppb) in 2021, > 160% higher than the pre-industrial level (year 1750). This increase is largely driven by anthropogenic activities (Skeie et al., 2023), like agriculture, waste management, fossil fuel extraction, and consumption, along with biomass combustion, natural emissions (wetlands and other inland aquatic environments) (Karoff & Vara-Vela, 2023; Mashiyi et al., 2024) and sinks (OH oxidation, other chemical reactions with chlorine and oxygen radicals, and soil uptake) (Stecher, 2024; Zhao et al., 2019). Therefore, lowering the concentrations of atmospheric CH_4_ is an effective option for addressing short-term climate change contribution to meet the goal of the Paris Agreement (Stecher, 2024).

Methane serves as a significant precursor for tropospheric ozone (Staniaszek et al., 2022). The primary sink of methane is through chemical reactions from the atmosphere (Skeie et al., 2023). CH_4_ influences atmospheric chemistry by regulating the levels of tropospheric hydroxyl radicals (OH) (Holmes, 2018). The atmospheric lifetime of OH is about ~ 1 s, and its concentration is reduced by the increased levels of methane, carbon monoxide (CO), and other compounds that have OH as their main atmospheric sink (Skeie et al., 2023). CO serves as a major reservoir (approximately 40%) for atmospheric OH radicals (Patel et al., 2024a). About 90% of tropospheric CH_4_ is removed by oxidation reaction with OH radical (Holmes, 2018; Skeie et al., 2023; Staniaszek et al., 2022; Zhao et al., 2019). CH_4_ oxidation results in the formation of water vapor (H_2_O) and ozone (O_3_), both of which significantly enhance effective radiative forcing, global warming potential, and the environmental risks (Holmes, 2018; Stecher, 2024). Tropospheric O_3_ poses harmful effects on human health and on vegetation (Stecher, 2024).

The lifespan of methane is highly influenced by its sources (Stecher, 2024) and fluctuations in CO levels; an increase in CO results in decreased OH levels in the atmosphere, thereby extending the lifetime of CH_4_, and vice-versa (Patel et al., 2024a). CH_4_ reduces tropospheric OH concentrations, which in turn extends the CH_4_ lifespan. This is referred to as the CH_4_ feedback effect (Holmes, 2018). Nitrogen oxides (NOx) play a significant role in atmospheric chemical cycling, affecting both O_3_ production and the secondary generation of OH through recycling processes. The reaction of nitrogen dioxide (NO_2_) competes directly with the reaction between CO and OH, as the CO–OH rate constant is considerably slower, yet CO exists in much greater concentrations. This scenario can be likened to substantial reductions in CH_4_ levels (Patel et al., 2024a, 2024b).

Botswana has experienced a notable upward trend in methane emissions. In 2019, the country was ranked 124th in methane emissions, with total emissions of 3.5 MtCO_2_e and a per capita emission of 1.46 MtCO_2_e (Climate Watch, 2025). There are several sources of methane emissions in Botswana including natural wetlands, livestock farming, and mining activities. (Helfter et al., 2022a, 2022b). Recent studies in Botswana highlight the role of wetland water systems and papyrus vegetation in controlling methane release especially in the Okavango Delta (Gondwe et al., 2021; Helfter et al., 2022a, 2022b; Lattaud et al., 2023). In contrast, the Central region is known for its mining activities, including diamond and coal extraction (Long, 2024), along with large livestock populations (Botswana, 2019), leading to significantly human-caused methane emissions (Janardanan et al., 2024; Sawyer et al., 2022). Apart from region specific methane sources, enteric fermentation in livestock, a natural digestive process also releases methane as a byproduct. Moreover, climate predictions suggest that less rainfall and higher temperatures, particularly in summer and autumn, could change methane emissions in the regions (Lachgar et al., 2022).

Despite evidence from existing research that suggests increasing methane levels and significant drivers in Botswana, there is limited current research providing direct, region-specific, measurement-based methane emission rates. Previous studies, particularly those conducted in the Okavango Delta wetlands, often rely on local measurements and mainly focus on these wetlands (Gondwe & Masamba, 2014; Helfter et al., 2020a, 2022a, 2022b; Lattaud et al., 2023; Masamba et al., 2015). The Central district lacks district-scale methane emission quantification and is only represented in national-scale satellite and model datasets (Janardanan et al., n.d.),(Janardanan et al., 2024). Satellite remote sensing, especially the European Space Agency’s Sentinel-5P TROPOMI instrument, provides high-resolution global data for monitoring methane (Lindqvist et al., 2024; Lorente et al., 2021; Zhang et al., 2023). However, the use of Sentinel-5P for thorough methane analysis in specific areas of Botswana is limited (Barré et al., 2021; Ouerghi et al., 2021; Schuit et al., 2023; Shikwambana et al., 2022; Trenchev et al., 2023a, 2023b). For instance, ground-based studies in the Okavango Delta have mainly focused on local methane fluxes (Gondwe et al., 2021; Helfter et al., 2020b; Lattaud et al., 2023). Gondwe et al. (2021) assessed methane emissions and environmental factors using closed chambers in a tropical swamp, while Helfter et al., (2020a, 2020b) reported 2 years of continuous methane measurements at two different wetland sites in the Okavango Delta. Additionally, Mfundisi et al. (n.d.) proposed a method for modeling methane emissions using MODIS data, concentrating on the flooding patterns in the Okavango Delta to enhance methane emission estimates. There is a significant gap in detailed, satellite-based studies specifically examine seasonal and long-term trends in methane across Botswana’s diverse regions.

To address these gaps, this study utilizes high-resolution Sentinel-5P satellite data to conduct a detailed, satellite-based assessment of methane’s seasonal and long-term trends in Botswana’s Central and Ngamiland regions. The study’s specific objectives of the study were to (1) generate high-resolution spatial distribution maps for identifying methane hotspots, (2) assess seasonal changes in methane concentrations by analyzing temporal trends, and (3) to apply statistical validation methods, including seasonal-trend decomposition (anomalies) and the Mann–Kendall test, to detect significant long-term trends over time. This study provides a practical and robust methane monitoring method applicable to future research in Botswana and other regions. This work support climate change mitigation efforts by providing actionable insights into methane emissions, enhancing the understanding of Botswana’s methane dynamics, distinguishing between natural and human influences, and paving the way for more focused mitigation strategies.

## Materials and methods

### Description of the study area

**Fig. 1:**
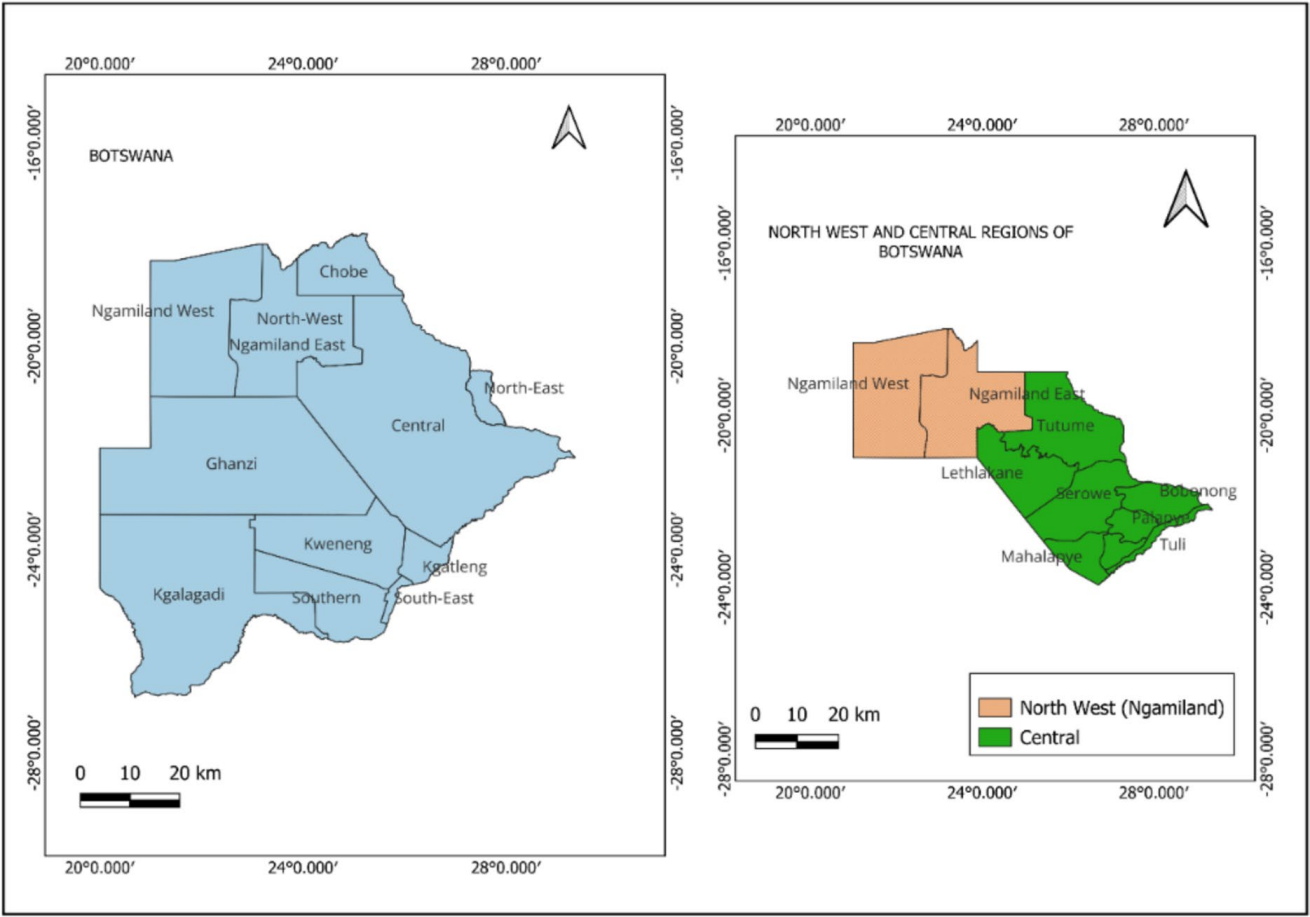
Study area map of Botswana, showing the Central and Ngamiland regions. These regions were chosen for their different methane emission profiles. The Central is marked by human-made sources, while Ngamiland features natural wetlands. Boundary shapefile data sourced from Diva-gis.org (https://www.Diva-gis.org/data; accessed: 10 December 2024)

The study focuses on two regions in Botswana: the Central district (21.5832° S, 26.0414° E) and the Ngamiland district (18.4256° S, 24.7143° E), as shown in Fig. 1. Figure 1 provides a map of Botswana that highlights these two study areas. Comparing emissions in Central and Ngamiland allows for a detailed look at CH_4_ distribution and its causes. These regions were selected because their CH_4_ emission profiles differ significantly, offering a good source to explore both natural and human contributions to atmospheric CH_4_ levels. The Central region is the largest administrative area in Botswana and includes a variety of landscapes and communities. It acts as a major population center (Nair & Masukusuku, 2013) and features a combination of rural and growing urban areas, creating a dynamic demographic (Barei, 2000; Buxton et al., 2023; Meyer-Emerick et al., 2004). The economy of the Central region is diverse, involving agriculture, mining and service industries. Coal mining is a key sector, especially in the Ngwato region (central) (Akintunde et al., 2023; Blackie et al., 2024).

### Ngamiland region

Ngamiland District is located in the northwestern Botswana and is marked by a variety of landscape, including pastoral areas, river systems, and large wildlife populations. This region is significant for its rural communities, which live near important water sources that affect land use, water needs, and local livelihoods (Gondo et al., 2023; Motho et al., 2022). People in Ngamiland rely on both traditional knowledge and modern methods to adapt to climate change and reduce disaster risks (Dintwa et al., 2022). The area has seen major changes in land use and land cover, with an increase in developed areas and a decrease in water and vegetation, raising concerns about sustainable development and environmental care (Gondo et al., 2023). Ngamiland is also an important center for community-based tourism. It makes use its natural resources and wildlife (Mbaiwa, 2018). The region faces challenges related to human-wildlife interactions, particularly with elephants, which pose risks to rural communities and require effective management strategies (Blackie et al., 2024).

## Data and methods

### Sentinel-5 precursor

Google Earth Engine (GEE), a cloud-based platform that allows for large-scale analysis of satellite data (Shikwambana et al., 2022) was used to process, analyze, and visualize Sentinel-5P methane concentration data. The Sentinel-5 Precursor (Sentinel-5P) satellite was launched in October 2017, which is part of the European Copernicus initiative (Jiang et al., 2024; Schneising et al., 2019; Shikwambana et al., 2022). Sentinel 5P holds the TROPOspheric Monitoring Instrument (TROPOMI), a highly advanced spectrometer (Ouerghi et al., 2021; Schuit et al., 2023) that has changed how methane is detected and improved the ability to track atmospheric CH_4_ concentration patterns and trends at high spatial resolution (Hossain, 2023; Maliehe, 2022; Schuit et al., 2023). The European Space Agency provides a methane dataset from Sentinel-5 in Earth Engine (Cardille et al., 2023). The satellite can cover the globe daily which enables continuous tracking of methane emissions. This is important for identifying trends and anomalies in methane concentration levels (Vanselow et al., 2024). This study collected data from the Sentinel-5P TROPOMI sensor (COPERNICUS/S5P/OFFL/L3_CH_4_) in the study regions from 2020 to 2023. A quality filtering process utilized the uncertainty band. This approach provides a reliable quantitative measure of data quality, as lower uncertainty values signify more dependable measurements. This dataset provides CH_4_ column volume mixing ratios in dry air expressed in parts per billion (ppb) and have a spatial resolution of 7 km by 7 km.

### Statistical analysis and data visualization

The descriptive statistics calculated included mean CH_4_ concentrations, seasonal-trend decomposition, and the Mann–Kendall test, to determine methane concentrations in Central and Ngamiland regions. Mean CH_4_ concentrations were calculated for each month and seasonal periods were Summer: December–February, Autumn: March–May, Winter: June–August, and Spring: September–November. Additionally, Mann–Kendall Tau (*τ*), a non-parametric test (Gadedjisso-Tossou et al., 2021; Sibiya et al., 2024) was used to determine statistically significant trends (*p* < 0.05) in the methane time series data. The *τ* statistic ranges from + 1 to − 1, this ranges explain whether there is a significant trend over time and whether the trends were upward or downwards. A value of + 1 indicates an increasing trend, while − 1 indicates a decreasing trend. Anomalies were detected monthly as deviations from the mean. The analysis focused on identifying positive anomalies that exceeded one standard deviation (> 1 *σ*) to highlight periods of unusually high methane emissions. Dynamic color-scaled concentration maps and charts were created using built-in tools from GEE and ArcGIS Pro. All statistical results and visual representations were systematically exported to Google drive for further analysis and interpretations.

## Results and discussions

### Methane concentration across study regions

**Fig. 2:**
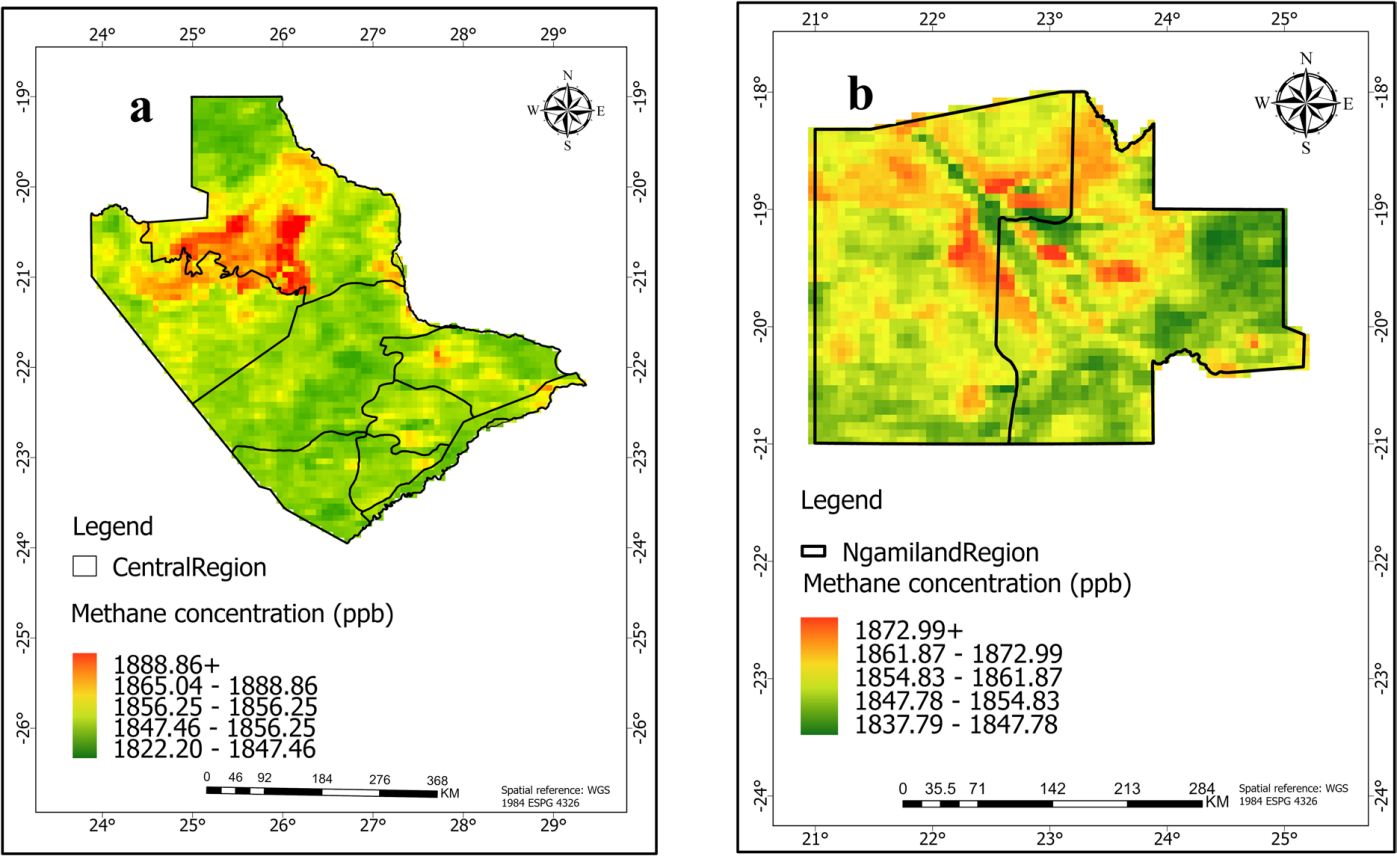
The mean methane concentration (ppb) from 2020 to 2023 in **a** Central and **b** Ngamiland regions of Botswana based on Sentinel-5P TROPOMI data

Figure 2 shows the distribution of mean methane concentrations across the central and Ngamiland regions. The color gradients represent concentration levels. The CH_4_ distribution varies in both regions, with mean concentrations ranging significantly from 1750 to 1900 ppb (Fig. 2). The variability suggests that local emissions strongly influence atmospheric CH_4_ levels. The Central region recorded minimum and maximum mean CH_4_ concentrations of 1818.11 ppb and 1873.05 ppb respectively, while Ngamiland recorded minimum of 1820.71 and maximum of 1876.38 ppb. These concentration levels are close to the global average. The National Oceanic and Atmospheric Administration (NOAA) recently reported global monthly average of 1925.99 ppb for February 2024 and 1933.97 ppb for February 2025. The latest update was on June 05, 2025 (Lan et al., n.d.). These levels highlight the region’s significant role in methane emissions. Hotspots, shown by warmer colors, are concentrated in densely populated or industrial areas, while cooler hues appear in rural or elevated regions.

In the Central region (Fig. 2a), average methane concentrations over the study period range from lower concentration of 1832.93 ppb to the highest concentration level of 1888.86 ppb. The highest values indicate hotspots or areas with significant methane emissions. There is clear spatial variability, with concentrations gradually decreasing from the central area outward. This pattern may suggest local sources such as agriculture, fossil fuel extraction, or human activities. The Central region has mines, including the Morupule coal mine, which may increase CH_4_ emissions from coal mining operations. Other mines in areas like the closed Selibe Phikwe and Orapa could also contribute to CH_4_ concentrations through degasification systems and ventilation systems. Based on the spatial trends, the Central region shows higher and more variable methane levels. Elevated concentrations are noticeable in the Letlhakane area, possibly linked to mining and pans, as well as in the Tutume, Serowe/Palapye areas, likely due to higher human activity like agriculture. For instance, the cattle population in Central Serowe/Palapye region is the highest, making up about 10.5% of the country’s total cattle population (Botswana, 2019) mainly due to the district’s large geographic area, compared to other regions.

In contrast, the Ngamiland region has slightly lower methane concentrations than the Central region, ranging from 1837.92 to 1872.99 ppb (Fig. 2b). While Ngamiland’s lower end (1837.92 ppb) is higher than Central’s (1832.93 ppb), Ngamiland’s upper end (1872.99 ppb) falls short of Central’s (1888.86 ppb). The smaller range in Ngamiland (about 35 ppb), compared to Central (approximately 56 ppb), indicates a more consistent distribution and less variability in Ngamiland. This narrower range indicates fewer differences in methane levels, likely due to more uniform land use or fewer human sources, suggesting less human impact. For instance, the Ngamiland district has the lowest recorded cattle population at 0.3% because of its small geographic area and limited livestock capacity (Botswana, 2019). As a result, methane emissions in this region may mainly come from natural sources such as wetlands like the Okavango Delta rather than human activities. Wetlands are an important natural source of methane. They contribute around 20–40% of global methane emissions (Wang et al., 1996). Changes in temperature and flooding patterns could greatly affect methane emissions from these wetlands (Gondwe et al., 2021; Helfter et al., 2020b; Masamba et al., 2015).

### Trend analysis: time series methane (CH4) concentration (ppb)

**Fig. 3:**
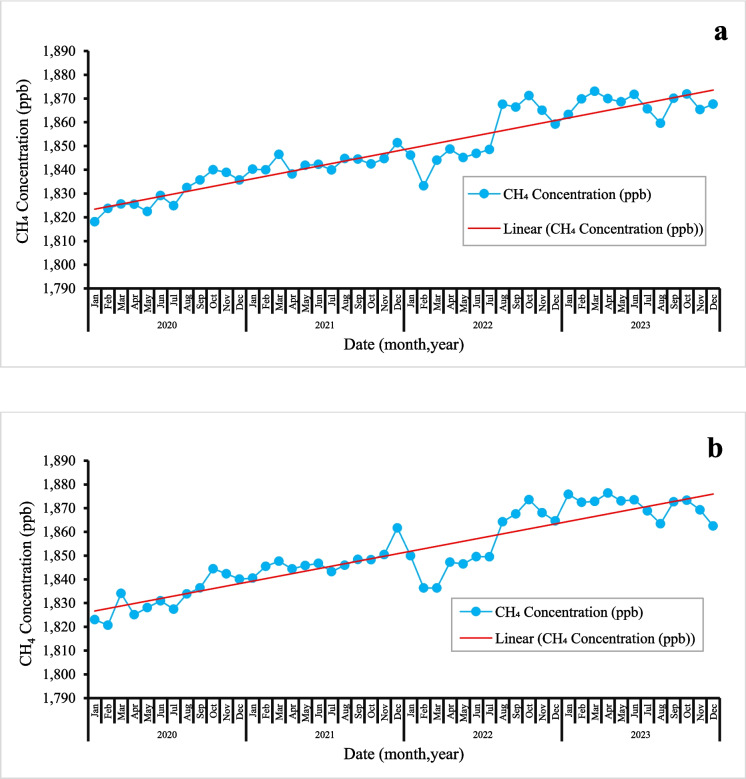
Time series of methane (CH_4_) concentrations (ppb) from 2020 to 2023 in **a** Central and **b** Ngamiland regions of Botswana. The blue points show the monthly average CH_4_ concentrations. The red line indicates the linear trend

Figure 3 displays line graphs illustrating the trends in CH_4_ concentrations (ppb) for the central (a) and Ngamiland (b) regions. Data points were recorded every month from 2020 to 2023. Methane concentrations show a slight linear increase over time, following the global trends of rising atmospheric levels. Although the line reflects seasonal changes, the overall upward trend is supported by time-series analysis (Fig. 3), showing a steady rise in methane emissions. However, both regions experienced a gradual decline in CH_4_ from February 2022 to July 2022 (Fig. 3), likely due to reduced natural effect especially seasonal pattern changes, since the decrease happened in both different regions over the same period. This period falls within the autumn and winter seasons. The seasons are cooler linked to reduced biological activity hence lower concentrations. In 2020, lower concentrations in methane emissions are linked to COVID lockdowns, whereby movement restrictions were enforced, reducing energy demand and limited maintenance during lockdown resulting to decrease in anthropogenic methane emissions (Skeie et al., 2023). The spikes in the Central region **(**Fig. 3a) may indicate an industrial influence of such mining activities. Furthermore, population growth in this region contributes to increased methane emissions. Higher population lead to more human activities, including increased agriculture and waste management challenges (Robinson et al., 2010). This growth raises the amount of solid waste, which means more landfill and dumping sites. The breakdown of this waste releases CH_4_ into the air (Gupta et al., 2022; Singh et al., 2025). Additionally, rising energy demands impact of electricity generation at the Morupule coal power station (Harvey, 2015), which may also increase methane emissions.

In contrast, the Ngamiland region particularly the Okavango Delta, features natural wetlands that are the main source of methane emissions **(**Fig. 3b). The time-series data for Ngamiland (Fig. 3b) indicate greater stability, suggesting that natural wetlands primarily driver methane levels. The stability means that the methane emissions in Ngamiland show a more uniform distribution and fewer sources, compared to the Central region, for example, Ngamiland has the lowest recorded cattle population (0.3%). While methane is consistently emitted from wetlands, the variability caused by human activities is less pronounced. Changes in temperature and waterlogged soils may be affecting the methane fluxes from the wetlands.

The time series observations indicate that these regions are hotspots for methane emissions, with an overall upward trend of about 5 ppb/year. This rate is higher than the global average of 3 ppb/year (Lan et al., n.d.). The trends align with the Mann–Kendall test results (Table 2), showing the statistically significant upward trends in both regions. The notable rise in methane levels, significantly above than the global average, suggests multiple factors are driving the increase in Botswana. These significant trends emphasize the need to address CH_4_ emissions in both areas. Tackling methane emissions requires identifying and addressing their root causes to effectively lower rising methane levels. This will help mitigate environmental and health risks while promoting sustainable development.

### Spatial and temporal patterns

**Fig. 4:**
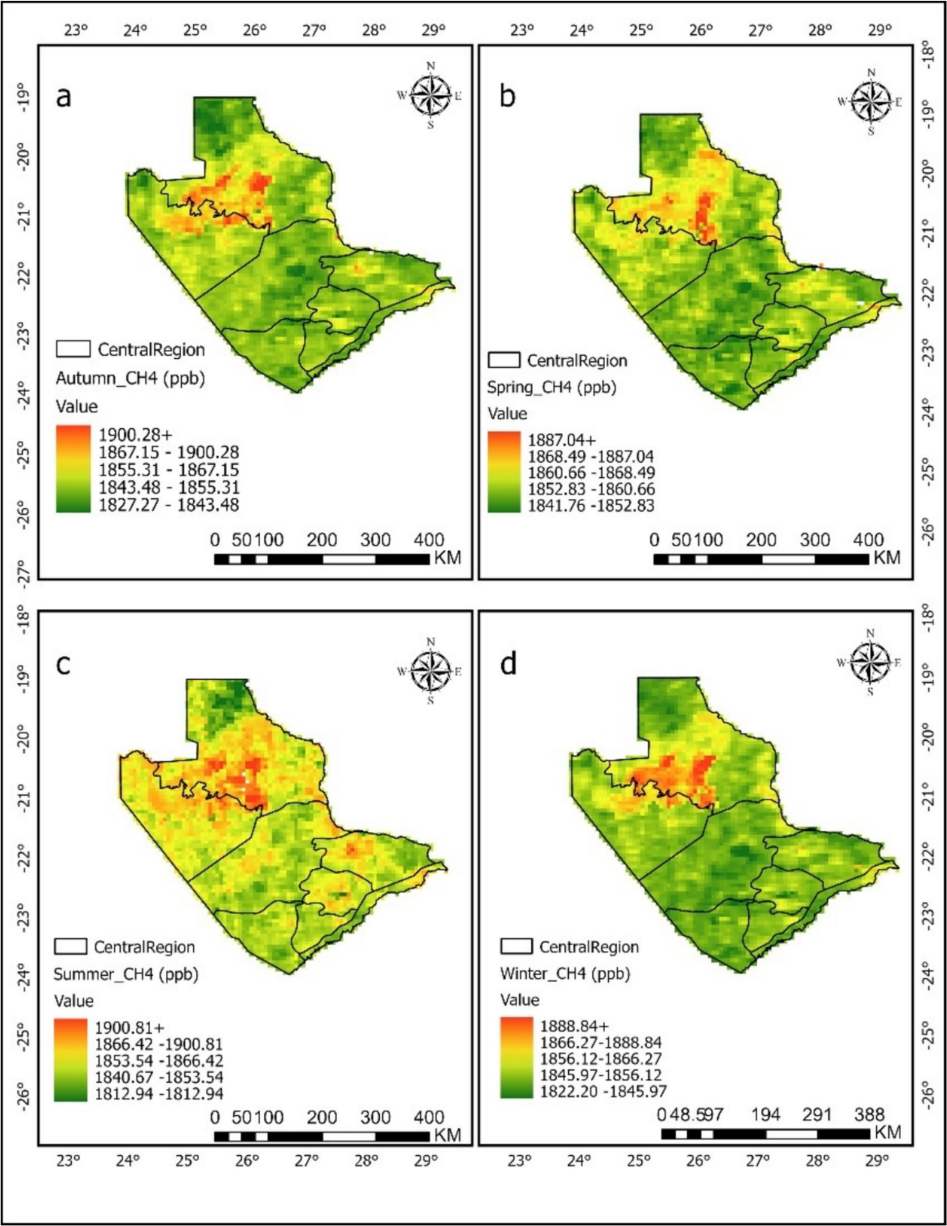
Seasonal methane (CH_4_) ppb levels in the Central region (2020–2023), showing spatial distribution for **a** autumn, **b** spring, **c** summer, and **d** winter. The maps highlight seasonal changes

**Fig. 5:**
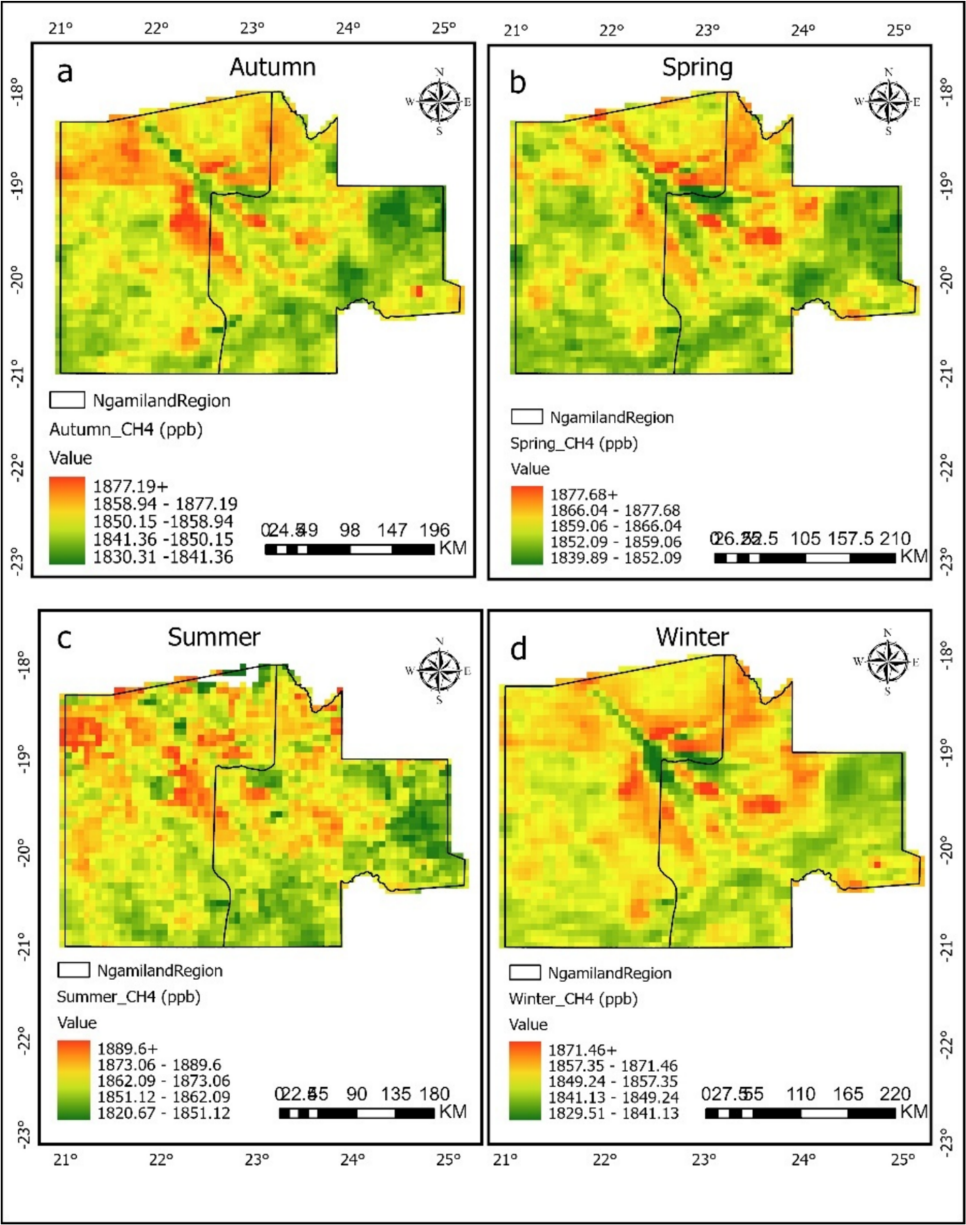
Seasonal methane (CH_4_) ppb levels in the Ngamiland region (2020–2023) show spatial distribution for **a** autumn, **b** spring, **c** summer, and **d** winter. The maps highlight seasonal changes

Figures 4 and 5 present seasonal maps of CH_4_ concentration (ppb) from 2020 to 2023 for autumn, spring, summer, and winter in the Central and Ngamiland regions, respectively. The figures highlight the differences in methane distribution over time. In the Central region (Fig. 4), the inset seasonal average shows that spring had the highest average concentration of 1855.40 ppb, followed by summer at 1852.17 ppb. Autumn and winter had CH_4_ averages of 1847.79 ppb and 1848.83 ppb, respectively (Table 1). The spring season in Botswana is represented by dry conditions, rising temperatures, and lower rainfall (Mberego, 2017). This may lead to increased biomass burning from wildfires, which raises methane emissions as noted by Makhado and Saidi (2020). The higher concentrations during spring might also come from temperature-driven emissions from landfills and agricultural activities when the weather conditions become warmer. The lower values seen in autumn and winter (Fig. 4) could be due to cooler temperatures (see Annex 2), which reduce biological activity and enhance atmospheric mixing (Berezina et al., 2023).

**Table 1:** Seasonal methane (CH_4_) ppb means across Central and Ngamiland regions (2020–2023)

Season	Central mean_CH_4_ (ppb)	Ngamiland mean_CH_4_ (ppb)
Summer	1852.17	1861.10
Autumn	1847.79	1849.39
Winter	1848.83	1849.49
Spring	1855.39	1857.55

In Ngamiland (Fig. 5), the seasonal methane trend is similar to that of Central region, but with slightly higher baseline levels in summer (1861.10 ppb), followed by Spring (1857.55 ppb), then autumn and winter at 1849.39 ppb and 1849.49 ppb, respectively. Autumn recorded the lowest concentration in the region (Table 1). The summer peak reaching up to 1889.60 ppb coincides with heavy flooding in the Okavango Delta. Additionally, Fig. 5 shows yellow to red hues around the Okavango Delta, which align with other studies indicating that wetlands have high methane concentrations (Gondwe et al., 2021; Helfter et al., 2020a, 2020b; Masamba et al., 2015). The higher emissions in summer may also be result from waterlogged soils, regardless of whether they are seasonally or permanently wet. Drier soils usually act as methane sinks due to microbial oxidation processes (Gondwe et al., 2021; Helfter et al., 2020b; Lattaud et al., 2023). Changes in precipitation patterns can affect waterlogged conditions, impacting methane levels as noted by Gondwe et al. (2021), who stated that soil water content is a key environmental factor controlling CH_4_ fluxes in both seasonal and occasional floodplains. Spring showed a rise in CH_4_ levels with the start of seasonal rains and wetland recharge, again tied to temperature and wet soils. High soil moisture is linked to increased emissions (Gondwe et al., 2021; Helfter et al., 2020b; Lattaud et al., 2023). Areas with seasonal flooding show lower emissions during dry periods and significant increases after rewetting events (Helfter et al., 2020a, 2020b). Areas that are frequently flooded or consistently dry tend to mainly act as methane sinks (Gondwe et al., 2021; Helfter et al., 2020b).

Various physical and chemical factors that influence methane are related to temperature changes (Masamba et al., 2015), which is supported by this study. However, the highest seasonal temperature recorded during the study occurred in spring, not summer (see Annex 2). Therefore, spring stands out as the main seasonal methane producer in Ngamiland region for all years (Table 1). The lower CH_4_ concentrations (Fig. 5) during cooler seasons likely result from reduced biological activity, similar to the central region (Fig. 4). Previous studies (Helfter et al., 2020a, 2020b; Lattaud et al., 2023) showed that methane emissions are lowest during dry periods. This confirms the findings of this study (Fig. 3). The results also align with the research by Gondwe and Masamba (2014), who found peak emissions (20–300 mg CH_4_ m^−2^ h^−1^) during warmer, rainy seasons, and minimal emissions (0.2–3.0 mg m^−2^ h^−1^) during cooler winter months. The relatively stable seasonal pattern in Ngamiland may suggest more consistent sources of emissions, like perennial wetlands. The studies about Okavango Delta of Ngamiland suggest that methane emissions are mainly influenced by soil moisture and temperature. Seasonal floodplains act as sources, while nearby dry soils serve as minor sinks.

### Spatial methane (CH4) anomalies and extreme events

**Fig. 6:**
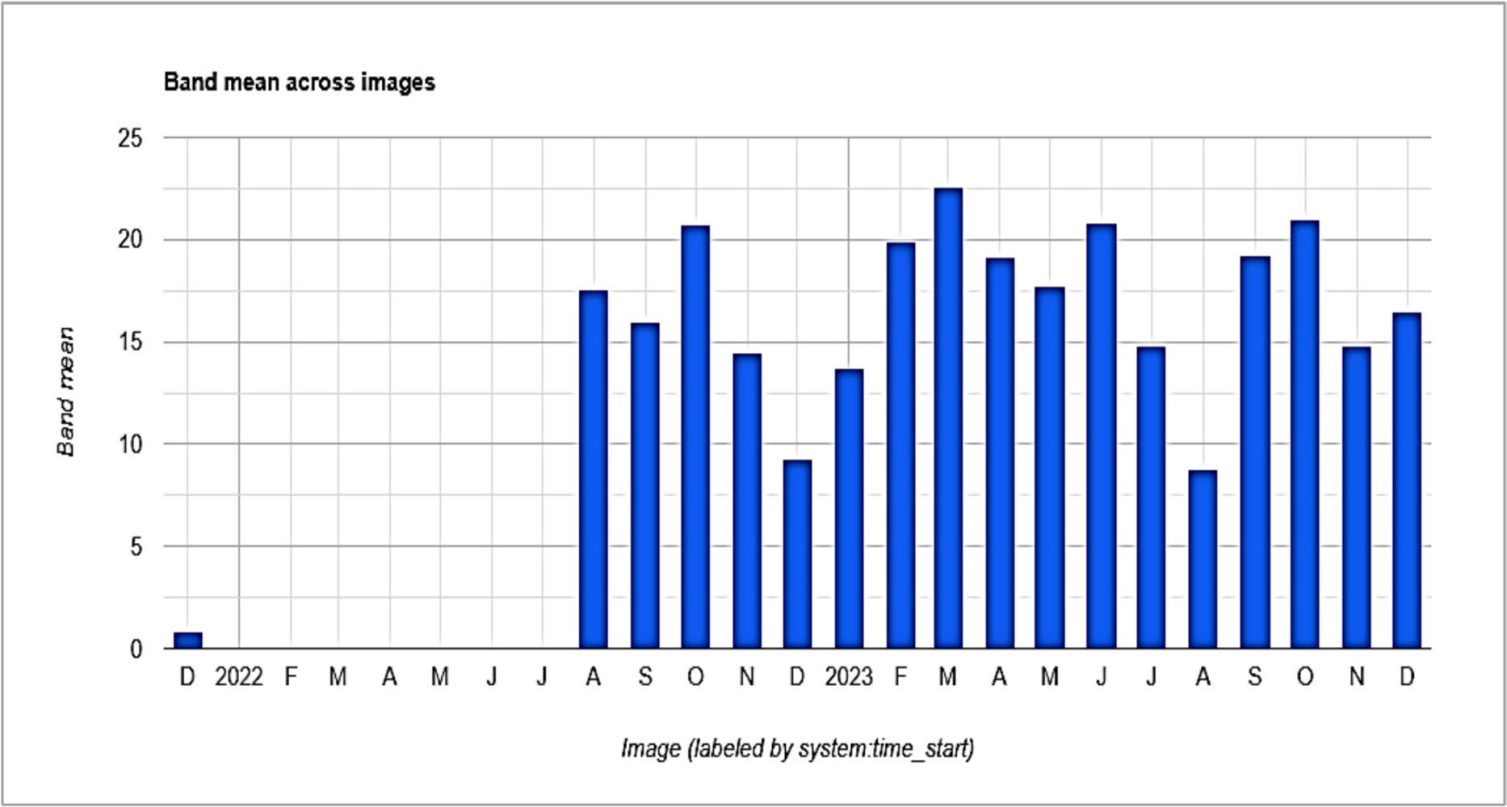
Monthly methane (CH_4_) anomalies (*σ*) in Central region (2020–2023). The bars show positive anomalies, indicating times of unusually high methane emissions above the long-term average. The gaps point to lower anomalies

**Fig. 7:**
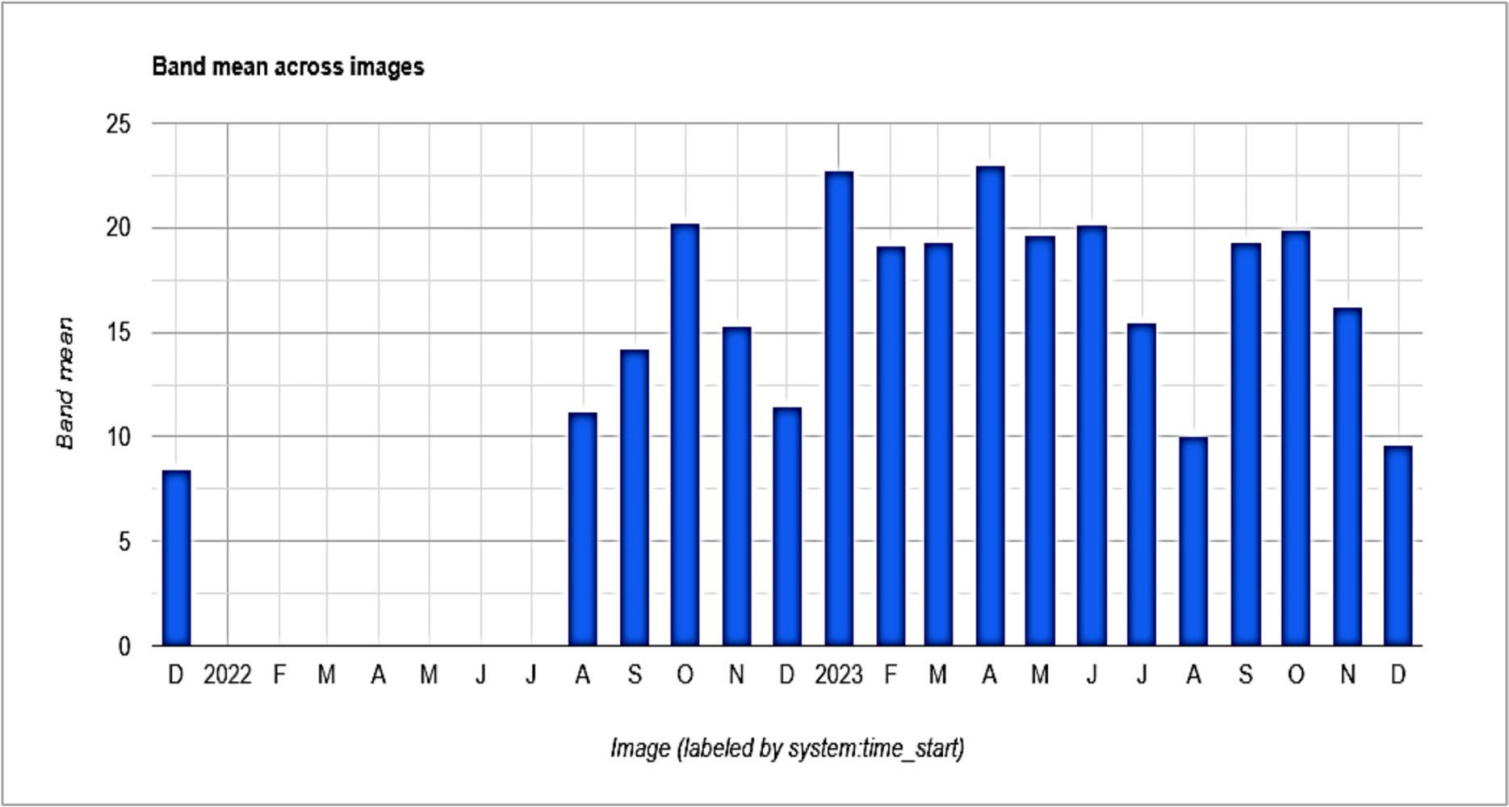
Monthly methane (CH_4_) anomalies (*σ*) in Ngamiland region (2020–2023). The bars show positive anomalies, indicating times of unusually high methane emissions above the long-term average. The gaps point to lower anomalies

The methane anomaly (*σ*) is a statistical measure that helps identify significant deviations in methane concentrations from their long-term average. It shows how much variation exists from the average methane emission levels. Anomaly analysis allows researchers to easily find specific dates or periods when methane concentrations rise significantly above expected levels (spikes or unusually high methane emissions) which might be missed in broader trend analyses. For instance, Fig. 3 shows the methane emission trend for the study period but does not clearly indicate spikes in methane levels. The figures below focus only on positive anomalies in methane concentrations within the Central (Fig. 6) and Ngamiland (Fig. 7) regions. Positive anomalies or values indicate the methane emissions exceeding the average levels, signaling increased methane-related risks. In instances where there is no data in Figs. 6 and 7 for a specific date, it means the values were negative, indicating methane emissions are below the long-term average and suggesting lower methane risk probabilities.

In both figures, from 2020 onwards, both regions showed negative anomalies except for December 2022. In December, Central recorded a value of 0.83 and Ngamiland recorded 8.46, but methane concentrations for both regions were the same (1840.13 ppb) on that date. The spike in methane levels may come from urban or industrial emissions, or sporadic natural releases. The spike seen in late 2021 across both regions could suggest a shared underlying cause, like a significant climatic event or a satellite artifact (Figs. 6 and 7). The higher positive anomalies observed in Ngamiland compared to Central suggest that methane risk in Ngamiland was greater. In Ngamiland (Fig. 7), the more significant positive anomalies may stem from stronger seasonal cycles that align with hydrological changes. Negative monthly anomalies (Figs. 6 and 7) suggest mitigation measures may have helped reduce emissions. From August 2021 until the end of the study period, both regions mainly showed values of + 1*σ*. It is important to note that despite the Botswana’s government National Adaptation Plan (NAP) and Action Plan developed in 2020, with various guidelines for climate change adaptation (Masocha et al., 2024), methane risks continued, and methane concentrations rose (Fig. 3).

Botswana faced frequent droughts, leading the government to declare the period of 2022/2023 as a severe drought period for agriculture. This was due to very low rainfall, extremely high temperatures, and long dry spells across the country (Masocha et al., 2024). Nevertheless, Ngamiland region showed a higher positive anomaly of 23 in April during this time, suggesting that the main contributors are not solely related to rainfall but may also include higher temperatures. Significantly, extreme positive anomalies (> 1.5 *σ*) in 2022 and 2023 were linked to above-average precipitation, indicating that hydrological extremes increase CH_4_ emissions from wetland sources. Around 12% of the months had positive CH_4_ anomalies (> 1 *σ* above the mean). The most significant anomalies were recorded in 2022 and 2023, aligning with extreme weather events like floods and droughts that may have disrupted established emission patterns.

### Trend analysis using Kendall’s tau (τ)

The findings from the Mann–Kendall test showed significant trends in CH_4_ concentrations in both the Central and Ngamiland regions of Botswana. In each case, the null hypothesis was rejected, which states that there is no trend in the dataset, based on the calculated *p*-values (Table 2). The Mann–Kendall test validated these significant upward CH_4_ concentration trends in both the Central (*p*-value = 6.6044e^−01^) and Ngamiland (*p*-value = 6.6159e^−01^) regions, as shown by the time-series analysis (Fig. 3). The statistically significant trends identified by the Mann–Kendall test and the positive anomalies (Figs. 6 and 7) since September 2022 suggest environmental risks. This highlights the importance of addressing CH_4_ emissions in both regions. This will help lower environmental and health risks while supporting sustainable development.

**Table 2:** Mann–Kendall test (τ) for CH_4_ concentrations in Central and Ngamiland regions; (*p*-value < 0.05)

Parameter	*p*-value	Null hypothesis (H0)
Central	6.6044e^−01^	Rejected, significant trend
Ngamiland	6.6159e^−01^	Rejected, significant trend

## Limitations and future work recommendation

### Limitations

The main limitation is the lack of direct flux data for validation in both regions. This means that it is crucial to conduct ground-based analysis of satellite-derived CH_4_ concentrations to correct possible biases. Lack of field-based CH_4_ flux measurements for different sources, such as terrestrial sinks outside Okavango, wildlife enteric fermentation, savanna fires, coal-bed, or geological sources, represents a significant gap. Botswana regions do not have long-term surface atmospheric monitoring, with much of the atmospheric CH_4_ concentration data derived from satellites. This makes it difficult to independently verify long-term trends using ground observations. Although Sentinel-5P has high-resolution capabilities, its spatial resolution of 7 km × 7 km can complicate the detection of small-scale emission sources. This requires combining satellite data with other datasets and improved analytical techniques.

### Future work recommendation

The spatial and temporal dynamics of methane emissions in Botswana highlight the need for further research to understand how these emissions vary across different regions. Future studies should focus on specific sources and use satellite data (Sentinel-5P data) alongside ground measurements and environmental modeling. Ground-based sensors can improve source identification and help reduce biases. This approach will provide a better understanding of methane dynamics in sensitive and diverse ecosystems. It will also support effective methane reduction strategies and offer detailed insights into what drives emissions. Integrated studies are vital for understanding the complex patterns of methane emissions in these regions. Research should investigate how land-use changes, such as deforestation and agricultural expansion, affect methane emissions. Exploring sustainable livestock management practices can help decrease methane emissions from agriculture. Additionally, future studies should use established satellite techniques with extensive multi-year datasets to improve understanding of methane emissions variability in different areas. Incorporating isotopic analysis is also advised to distinguish between biogenic sources like wetlands, and human-made sources, such as agriculture and fossil fuels. This will help in identifying sources more accurately. Future efforts should include targeted policy measures for emission controls and focus on monitoring seasonal changes.

## Conclusion

The study successfully used Sentinel-5P satellite data to identify methane emission patterns and trends in Botswana. It proved to be a valuable monitoring tool. The study showed a rise in methane levels, with clear seasonal changes. For instance, Ngamiland had higher methane in summer related to increased flooding and microbial activity in its wetlands. This shows how natural processes greatly contribute to methane production in Ngamiland. Meanwhile, the emissions in the Central region come from both natural and human influences. The study also found specific high-emission hotspots. In the Central region, these are linked to industrial and urban development. In Ngamiland, they mainly result from natural wetland processes and organic decomposition. The Mann–Kendall test confirmed that these increases are statistically significant and represent long-term changes, not just random variations. An ongoing rise in methane could worsen global warming and create serious environmental and health risks. Thus, this study recommends region-specific policy measure. In the Central district, the focus should be on fixing industrial leaks and improving urban emission controls. Ngamiland region should focus on improving wetland conservation and monitoring. Further research should combine satellite data with the stated recommendations. This combined approach will improve understanding of methane sources and offer more detailed insights for effective mitigation. In summary, this study provides a clear, practical framework for monitoring methane emissions. The framework will enable local authorities and policymakers to tackle climate change challenges by refining observation techniques and accurately identifying methane sources.

## Supplementary Information

Below is the link to the electronic supplementary material.

Supplementary file1 (DOCX 80.2 KB)

## Data Availability

No datasets were generated or analysed during the current study.

## Declarations

All authors have read, understood and have complied as applicable with the statement on “Ethical responsibilities of Authors”, as found in the Instructions for Authors.
