# Supplementary material for: Satellite-driven assessment of methane trends, seasonal variability, and emission hotspots in Botswana’s Central and Ngamiland Regions
Source: Environ Monit Assess. 2025 Sep 24;197(10):1143. doi: 10.1007/s10661-025-14609-y (PMC12460582; doi:10.1007/s10661-025-14609-y)
Supplement: Supplementary file 1 — Supplementary file1 (DOCX 80.2 KB) [file 10661_2025_14609_MOESM1_ESM.docx]

**Annexes**

**Annex 1:** Average Precipitation (Avg. precipitation (mm/day)), Maximum Temperature (^o^c) and Minimum Temperature (^o^c) of the **Central region** for the year 2020 to 2023

**Annex 2:** Seasonal Average Precipitation (Avg. precipitation (mm/day)), Maximum Temperature (^o^c) and Minimum Temperature (^o^c) of the **Ngamiland region** for the year 2020 to 2023

***Source:*** *NASA Power DAV v2.5.0.* [*https://power.larc.nasa.gov/data-access-viewer/*](https://power.larc.nasa.gov/data-access-viewer/)*, United States government (accessed 27/07/2025).*
